# In Vitro Assessment of the Prebiotic Effects of Poria Cocos Polysaccharides Using Fecal Microbiota from Normal-Weight and Obese Children

**DOI:** 10.3390/foods14234077

**Published:** 2025-11-27

**Authors:** Dan-Yi Qiu, Xiao-Qin Liu, Yue Luo, Xin Chen, Wen-Na Zhang, Xin Yang, Fei-Hong Luo, Rui-Rui Wang

**Affiliations:** 1 Department of Pediatric Endocrinology and Inherited Metabolic Diseases, Children’s Hospital of Fudan University, Shanghai 201102, China; 23211240016@m.fudan.edu.cn; 2 Shanghai Innovation Center of TCM Health Service, Shanghai University of Traditional Chinese Medicine, Shanghai 201203, China; liuxiaoqin0528@163.com (X.-Q.L.); 22024646@shutcm.edu.cn (Y.L.); chenxin110595@163.com (X.C.); wennazhang24128@163.com (W.-N.Z.); yang.xin@yale.edu (X.Y.); 3 State Key Laboratory of Integration and Innovation of Classic Formula and Modern Chinese Medicine, Shanghai University of Traditional Chinese Medicine, Shanghai 201203, China; 4 Section of Endocrinology, Internal Medicine, School of Medicine, Yale University, New Haven, CT 06510, USA

**Keywords:** Poria cocos, in vitro fermentation, microbiota, short-chain fatty acids (SCFAs), indolelactic acid

## Abstract

Poria cocos polysaccharides (PCP) are recognized as potential prebiotics with documented metabolic benefits in adults. However, their impact on the gut microbiota of children remains unclear. This study aimed to evaluate the effects of PCP versus inulin (INL) on the gut microbiota and bacterial metabolites in normal-weight children (CON) and obese children (OB). In vitro fermentation was conducted using fecal samples pooled from five normal-weight children and five obese children, respectively. The samples were incubated with PCP, INL, or a blank control under anaerobic conditions at 37 °C for 24 h. After fermentation, the effects of PCP and INL on gut microbiota were evaluated using 16S rRNA sequencing. Bacteria-derived metabolites were measured using targeted metabolic profiling. Single-strain validation was performed to confirm effects on key bacterial taxa. PCP supplementation promoted the growth of *Bifidobacterium* and *Limosilactobacillus* in both normal-weight and obese children, accompanied by a rise in acetic acid production, particularly in normal-weight children. Compared to INL, PCP showed similar but slightly weaker effects on *Bifidobacterium* growth and short-chain fatty acids (SCFAs) production, but more strongly stimulated *Limosilactobacillus* growth. Notably, PCP also stimulated the production of indolelactic acid in both obese and normal-weight children. Correlation analysis indicated that *Bifidobacterium* and *Limosilactobacillus* were positively associated with acetic acid, lactic acid, and indolelactic acid, and negatively associated with tryptophan. Single-strain fermentation supported the community-level findings. PCP and INL both modulate gut microbiota and metabolic profiles in children, with PCP demonstrating a distinct prebiotic profile. Notably, PCP increased health-associated metabolites such as acetic acid and indolelactic acid, which are implicated in gut barrier support, immune modulation, and metabolic regulation. These findings suggest PCP may be considered a functional food component for supporting gut health in children, warranting further research.

## 1. Introduction

Wolfiporia extensa (syn. *Poria cocos*), known as “Fuling” in China, is a fungal species widely used in functional foods and traditional Chinese medicine [1]. Studies have demonstrated that Wolfiporia extensa is rich in polysaccharides and triterpenoids [2,3]. Poria cocos polysaccharides (PCP) are the principal constituents of Wolfiporia extensa, constituting approximately 70–90% of the dry sclerotial biomass [4,5]. Further studies have revealed that PCP have broad biological activities, such as anti-inflammatory, immunomodulatory, antioxidant, tumor suppression, and organ protection, particularly in the liver and kidney [3,4,6,7,8].

Gut microbiota, composed of trillions of microbes in the human colon, plays an important role in maintaining host homeostasis [9]. The gut microbiota generates a wide range of metabolites of multiple chemical categories, including lipopolysaccharides, short-chain fatty acids (SCFAs), bile acids, tryptophan metabolites, and indole derivatives [10]. They act as key molecular mediators that link microbial activities to host metabolism, immunity, and disease [11]. Recent studies have indicated that active components of functional foods could modulate the composition and function of gut microbiota to maintain metabolic health [10,12,13,14]. For example, Ge et al. [15] reported that polysaccharides from coix seed regulated the composition of gut microbiota by increasing the relative abundance of *Bifidobacterium*, *Limosilactobacillus*, and *Collinsella*. In addition, these polysaccharides also facilitated the production of SCFAs.

The prevalence of childhood obesity has increased rapidly in recent years, representing a major global public health challenge [16]. Accumulating evidence indicates that dietary factors influence childhood obesity both directly and indirectly, through the modulation of gut microbiota, which plays an important role in obesity development [17,18]. Compared to adults, children exhibit distinct gut microbiota profiles [19]. According to Radjabzadeh et al. [20], children had notably lower diversity than adults. Additionally, they observed a higher relative abundance of the genus *Bacteroides* in children, while the genus *Blautia* was more abundant among adults. These developmental differences in key genera mean that findings from adult studies cannot be directly extrapolated to pediatric populations. In addition, obese children show significant alterations in gut microbiota relative to their normal-weight peers [17]. Previous studies have shown that PCP can enhance the relative abundance of beneficial bacteria, such as *Lactobacillus* and *Bifidobacterium*, as well as increase SCFA concentrations in normal-weight adults [2]. Nevertheless, the effects of PCP on the gut microbiota of children, especially those with obesity, have not yet been reported.

Here, we outline the experimental design, rationale, and protocols developed for the in vitro fermentation study assessing the effects of PCP and inulin (INL) on the gut microbiota and metabolic profiles of children. The study utilized fecal samples from two distinct groups: normal-weight children (CON) and obese children (OB). To our knowledge, this study is the first to examine how PCP influence the gut microbiota and metabolites of obese children. The findings are anticipated to provide important evidence for the potential use of PCP in child-oriented functional foods.

## 2. Materials and Methods

### 2.1. Children’s Research Subjects

Ten children, consisting of 5 normal-weight children and 5 obese children, were enrolled. Obesity was defined according to the age- and sex-specific BMI reference cutoffs for overweight and obesity in Chinese children and adolescents (2–18 years), which were recommended by the Working Group on Obesity in China (WGOC) [21]. Subjects were excluded from taking antibiotics, prebiotics, or other microbiota-modulating drugs within one month. Subjects with acute or chronic gastrointestinal diseases were also not enrolled.

### 2.2. Laboratory Measurements

In this study, participants’ FBG, FINS, ALT, AST, SCR, UA, TC, TG, HDL-C and LDL-C were measured in the Clinical Laboratory of the Children’s Hospital of Fudan University.

### 2.3. Materials and Equipment

A commercial water-soluble PCP product (80% purity, S25272) was obtained from Yuanye Biotechnology Co., Ltd., Shanghai, China. INL was procured from Fuxuan Biotechnology Co., Ltd., Jinhua, Zhejiang, China. MPYG medium (HB8931), MRS medium (HB0384-1), anaerobic packages (HBYY001), and anaerobic bags (HBYY007) were purchased from Hopebio, Qingdao, Shandong, China. L-cysteine was obtained from Damas-Beta, Shanghai, China. The anaerobic workstation (Concept 400) was obtained from The Baker Company, Sanford, ME, USA. The refrigerated centrifuge (Z216MK) was procured from Hermle, Wehingen, BW, Germany. The Stratus plate reader was purchased from Cerillo, Charlottesville, VA, USA.

### 2.4. Monosaccharide Analysis of PCP

Monosaccharide composition was determined by trifluoroacetic acid (TFA; ANPEL, Shanghai, China) hydrolysis followed by ion chromatography. PCP samples were hydrolyzed with 2 M TFA at 121 °C for 2 h. Samples were dried under a nitrogen stream, washed with methanol (ANPEL), and evaporated to dryness. The washing procedure was repeated 2–3 times. The residue was dissolved in deionized water and filtered through 0.22-μm microporous filter for measurement.

The sample extracts were analyzed by high-performance anion-exchange chromatography with pulsed amperometric detection on a Dionex ICS-5000+ system (Thermo Fisher Scientific, Waltham, MA, USA). Separation was carried out on a CarboPac™ PA20 column (3 × 150 mm, 10 μm; Dionex, Sunnyvale, CA, USA) with a flow rate of 0.5 mL/min and an injection volume of 5 μL. The mobile phases consisted of deionized water (A), 0.1 M NaOH (B), and 0.1 M NaOH (Sigma-Aldrich, St. Louis, MO, USA) containing 0.2 M sodium acetate (Sigma-Aldrich) (C). The gradient program was as follows: 0–26 min, a linear increase from A/B/C = 95/5/0 (*v*/*v*) to 85/5/10 (*v*/*v*); 26–42 min, held at 85/5/10 (*v*/*v*); 42.0–42.1 min, changed to 60/0/40 (*v*/*v*); 42.1–52.0 min, maintained at 60/40/0 (*v*/*v*); 52.0–52.1 min, returned to 95/5/0 (*v*/*v*); and 52.1–60 min, equilibrated at 95/5/0 (*v*/*v*). Monosaccharides were identified by comparing retention times with authentic standards (Sigma-Aldrich), and quantification was performed using calibration curves derived from standard solutions. Chromatographic data were collected and processed using Chromeleon 7.2 CDS software (Thermo Fisher Scientific), and quantitative results were exported for further statistical analysis.

### 2.5. Collection of Fecal Samples and In Vitro Fermentation

Fecal samples from normal-weight and obese children were collected and immediately placed into 5 mL sterile cryogenic storage tubes, which were then transferred into anaerobic bags containing pre-anaerated packages. The samples were subsequently stored at −80 °C until further use for in vitro fermentation. Frozen fecal samples were thawed on ice, and fecal samples from children in the CON and OB groups were pooled separately to reduce inter-individual variation. The pooled samples were then mixed with phosphate-buffered saline (pH 7.2) to prepare a 10% (*w*/*v*) suspension, and subsequently vortexed until no visible large fecal particles were seen. The mixture was centrifuged at 2000× *g* for 10 min. The supernatants were transferred into sterile centrifuge tubes.

Previous studies have demonstrated that a concentration of 1% (*w*/*v*) PCP and INL is commonly used in in vitro fermentation models to ensure sufficient substrate to assess gut microbiota activity [2,22]. Accordingly, fermentation was carried out in MPYG medium with either PCP or INL at 1% (*w*/*v*) to establish the PCP group and the INL group. The MYPG medium without PCP or INL served as the blank group (BLK group). Each medium (PCP-containing, INL-containing, or BLK) was then mixed with fecal suspension at a ratio of 19:1 (*v*/*v*) and incubated at 37 °C for 24 h. After centrifugation at 9000× *g* for 5 min, the supernatants were filtered through a 0.22-μm membrane. Both supernatants and sediments were preserved at −80 °C until use.

### 2.6. In Vitro Culture of Single Strains

*Bifidobacterium pseudocatenulatum* ATCC27919, *Limosilactobacillus reuteri* ATCC23272, and *Enterococcus faecium* ATCC19433 were purchased from Biobw Biotechnology Co., Ltd., Beijing, China. These strains were activated in MRS broth containing 0.05% L-cysteine at 37 °C for 24 h. Following activation, the cultures were divided into three groups based on supplementation: the PCP group (MRS supplemented with 1% [*v*/*v*] PCP), the INL group (MRS supplemented with 1% [*v*/*v*] INL), and the BLK group (MRS without supplementation, serving as the blank control). After 24 h of co-culture, the optical density at 600 nm (OD600) was measured.

### 2.7. DNA Extraction,16S rRNA Gene Sequencing

Microbial DNA was extracted from samples using the FastPure Stool DNA Isolation Kit (MJYH, Shanghai, China). DNA integrity and concentration were verified by agarose gel electrophoresis and NanoDrop 2000 (Thermo Fisher Scientific). The V3–V4 region of the 16S rRNA gene was amplified using TransStart Fastpfu DNA Polymerase (AP221-02, TransGen Biotech, Beijing, China). The amplification employed 338F (5′-ACTCCTACGGGAGGCAGCAG-3′) and 806R (5′-GGACTACHVGGGTWTCTAAT-3′) primer pairs on an ABI GeneAmp^®^ 9700 Thermal Cycler (Applied Biosystems, Foster City, CA, USA). PCR products were purified using the PCR Clean-Up Kit (YuHua, Shanghai, China), quantified using Qubit 4.0 (Thermo Fisher Scientific). Purified amplicons were pooled in equimolar amounts and sequenced on the Illumina NextSeq 2000 platform (Illumina, San Diego, CA, USA) by Majorbio Bio-Pharm Technology Co., Ltd. (Shanghai, China).

Raw reads were processed with fastp (v0.19.6), where reads were trimmed when the average quality score fell below Q20 within a 50 bp sliding window, and reads <50 bp or containing ambiguous nucleotides were removed. The resulting high-quality reads were then merged using FLASH (v1.2.7). High-quality sequences were clustered into operational taxonomic units (OTUs) at a 97% similarity threshold with UPARSE (v7.1). Taxonomic classification of representative sequences was conducted with the RDP Classifier (v2.2) against the SILVA v138 database, applying a confidence cutoff of 0.7. To standardize sequencing depth across samples, all datasets were rarefied to 20,000 reads, with Good’s coverage exceeding 99%.

### 2.8. Targeted Metabolomic Profiling

Supernatants from fermentation were thawed on ice, lyophilized to remove residual water, and then transferred to the Eppendorf epMotion Workstation (Eppendorf Inc., Hamburg, Germany). A total of 120 μL of ice-cold methanol (Thermo Fisher Scientific) containing partial internal standards was automatically added to each sample. The mixture was vortexed thoroughly and then centrifuged at 4000× *g* for 30 min. The clear supernatants were returned back to the workstation. Then, 30 μL of the supernatant was transferred to a clean 96-well plate, and 20 μL of freshly prepared derivatization reagents (Merck KGaA, Darmstadt, Germany) was added to each well. After derivatization, 330 μL of ice-cold methanol was added to dilute the samples. The samples were then stored at −20 °C for 20 min and centrifuged again at 4000× *g* at 4 °C for 30 min before analysis.

Metabolite analysis was performed on an ACQUITY UPLC–MS/MS system (Waters Corp., Milford, MA, USA) equipped with a BEH C18 analytical column (2.1 × 100 mm, 1.7 μm; Waters Corp.) and a VanGuard pre-column (2.1 × 5 mm, 1.7 μm; Waters Corp.). The column and auto-sampler temperatures were maintained at 40 °C and 10 °C, respectively. The flow rate was set to 0.4 mL/min, and the injection volume was 5 μL. Mobile phases consisted of water with 0.1% formic acid (Sigma-Aldrich) (A) and acetonitrile/isopropanol (Thermo Fisher Scientific) (70:30, *v*/*v*) (B), with a gradient elution program as follows: 0–1 min (5% B), 1–11 min (5–78% B), 11–13.5 min (78–95% B), 13.5–14 min (95–100% B), 14–16 min (100% B), 16–16.1 min (100–5% B), and 16.1–18 min (5% B).

Raw UPLC-MS/MS data were analyzed using the TMBQ software (v1.0, Metabo-Profile, Shanghai, China) to perform peak identification, integration, calibration, and quantitation for each metabolite [20].

### 2.9. Statistical Analysis of Data

All data were presented as the mean ± standard deviation (SD). Statistical analyses were performed using GraphPad Prism software (v10.1.2, USA) and IBM SPSS Statistics (v25, USA). For comparisons between two groups, Student’s *t*-test or the Mann–Whitney U test was applied depending on data distribution. For three groups, two-way ANOVA was used, followed by Tukey’s post hoc test. Statistical significance was established at the thresholds of *p* < 0.05 (*), *p* < 0.01 (**), and *p* < 0.001 (***). Bioinformatic analysis was conducted using the OECloud platform (https://cloud.oebiotech.com, accessed on 28 September 2025; OE Biotech, Shanghai, China).

## 3. Results

### 3.1. Demographic and Clinical Characteristics of Subjects

The demographic and clinical characteristics of the participants are listed in Table S1. The age and sex distributions were comparable between the two groups. In comparison to the CON group, the OB group exhibited significantly higher BMIs (*p* < 0.01). In addition, ALT and TG levels were significantly elevated in the OB group (*p* < 0.01) (Table S1).

### 3.2. Monosaccharide Composition of Poria Cocos Polysaccharides

The structural features of PCP, including monosaccharide composition, molecular weight, chain conformation, glycosyl linkage type, and branch structure, have been extensively characterized in published literature [23,24]. Given this established body of structural knowledge, the analytical determination performed in the current study on the commercial PCP product focused on verifying its identity and quality control. This was achieved by determining the monosaccharide composition using ion chromatography. The result showed that PCP was mainly composed of four monosaccharide constituents, among which glucose was the primary constituent, accounting for approximately 89.74% of the total sugar content. The other identified components included fucose, galactose, and mannose (Figure S1 and Table S2). This result verified that the commercial product possessed the characteristic saccharide profile established in the literature for PCP [23,24].

### 3.3. Effect of PCP on the Gut Microbiota of Normal-Weight and Obese Children

**Figure 1:**
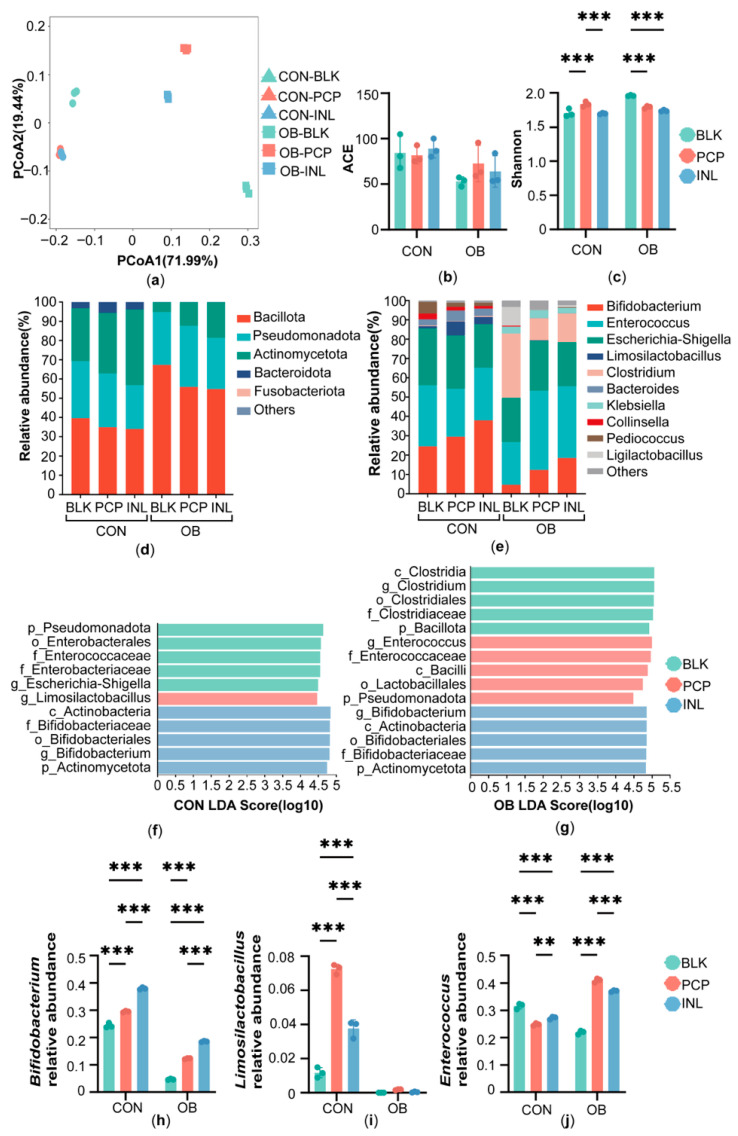
Gut microbiota structure after in vitro fermentation. (**a**) Principal coordinate analysis (PCoA) based on Bray–Curtis distances of the gut microbiota at the OTU level. Alpha diversity measured by (**b**) ACE (Abundance-based Coverage Estimator) index and (**c**) Shannon index. Gut microbiota composition at the (**d**) phylum and (**e**) genus level. Histograms illustrating the distribution of taxa identified through Linear Discriminant Analysis Effect Size (LEfSe) at two groups: (**f**) CON group and (**g**) OB group. Relative abundance of key genera: (**h**) *Bifidobacterium*, (**i**) *Limosilactobacillus*, and (**j**) *Enterococcus*. Data are expressed as the mean ± SD (n = 3), ** *p* < 0.01, *** *p* < 0.001.

In this study, 16S rRNA gene sequencing was used to assess the impact of PCP on the gut microbiota of normal-weight and obese children. Alpha diversity analysis of the fermentation system was assessed and presented using ACE (Abundance-based Coverage Estimator) and Shannon indices in Figure 1. In the OB group, PCP treatment increased the ACE index, but there were no statistically significant differences in either the CON or OB group (Figure 1b). Shannon diversity index increased significantly in the CON group but decreased in the OB group after PCP treatment (*p* < 0.001) (Figure 1c). Principal coordinate analysis (PCoA) based on Bray–Curtis distances was performed in Figure 1a. The first two principal components explained 71.99% and 19.44% of the variance, respectively (Figure 1a). The PCoA results showed that the CON and OB groups were well separated, indicating distinct microbial community structures between the CON and OB groups. In addition, in both the CON and OB groups, PCP and INL treatments were separated from BLK, indicating that PCP and INL treatments shaped the gut microbiota structure.

To further characterize these differences, we analyzed the bacterial composition of each group at the phylum and genus levels. At the phylum level, Bacillota, Pseudomonadota, Actinomycetota, Bacteroidota dominated after fermentation. Compared to BLK, PCP, and INL treatments increased the ratio of Actinomycetota and decreased ratio of Bacillota in both the CON and OB groups. INL treatment showed a greater increase in Actinomycetota than PCP treatment (Figure 1d). At the genus level, the microbial community after fermentation was predominantly composed of *Bifidobacterium*, *Enterococcus*, *Escherichia-Shigella*, and *Limosilactobacillus* (Figure 1e).

Linear Discriminant Analysis Effect Size (LEfSe) analysis was then conducted separately in the CON and OB groups (Figure 1f–g). *Bifidobacterium* was consistently enriched in INL treatment under both the CON and OB groups. *Limosilactobacillus* was specifically enriched in PCP treatment of the CON group, whereas PCP treatment in the OB group showed enrichment of the related order Lactobacillales. In addition, the BLK of the CON group was characterized by enrichment of the family Enterococcaceae. In contrast, in the OB group, PCP treatment promoted the enrichment of *Enterococcus* together with Enterococcaceae. Relative abundance analysis was consistent with the LEfSe analysis (Figure 1h–j). In the CON group, PCP treatment and INL treatment significantly increased the relative abundance of *Bifidobacterium* and *Limosilactobacillus* (*p* < 0.001). INL treatment showed a higher increase in the relative abundance of *Bifidobacterium*, whereas PCP treatment elevated the relative abundance of *Limosilactobacillus* (*p* < 0.001). In addition, PCP and INL treatments both significantly decreased the relative abundance of *Enterococcus* (*p* < 0.001). In the OB group, PCP and INL treatments increased the relative abundance of *Bifidobacterium* and *Limosilactobacillus*. However, compared to BLK, both PCP and INL increased the relative abundance of *Enterococcus* in the OB group (*p* < 0.001).

### 3.4. Impact of PCP on Targeted Metabolites

**Figure 2:**
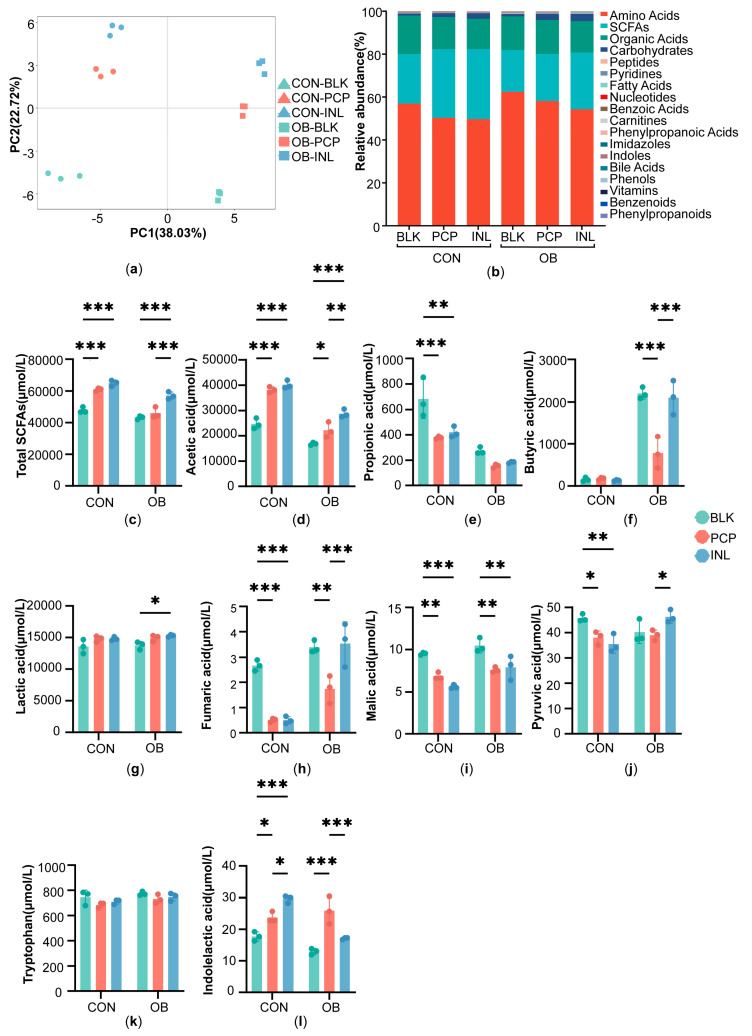
Metabolic profiles after in vitro fermentation. (**a**) Partial least squares discriminant analysis (PLS-DA) of metabolites. (**b**) Overall metabolite composition at the class level. The levels of (**c**) total SCFA production (the total amount of acetic acid, ethylmethylacetic acid, propionic acid, butyric acid, isobutyric acid, valeric acid, isovaleric acid, caproic acid and isocaproic acid), (**d**) acetic acid, (**e**) propionic acid, (**f**) butyric acid, (**g**) lactic acid, (**h**) fumaric acid, (**i**) malic acid, (**j**) pyruvic acid, (**k**) tryptophan, (**l**) indolelactic acid. Data are expressed as the mean ± SD (n = 3), * *p* < 0.05, ** *p* < 0.01, *** *p* < 0.001.

Since shifts in gut microbes are often associated with metabolic changes, we used targeted profiling of about 300 metabolites to assess the PCP’s functional consequences. Partial least squares discriminant analysis (PLS-DA) revealed a clear separation between the CON and OB groups. In both the CON and OB groups, PCP and INL treatments were well separated from BLK, indicating that PCP and INL could affect microbiota metabolism notably (Figure 2a). The detected metabolites mainly consisted of amino acids and their derivatives, SCFAs, organic acids, and carbohydrates (Figure 2b).

The results of SCFAs after fermentation are presented in Figure 2c–f. In the CON group, PCP and INL treatments raised the acetic acid and total SCFAs compared to BLK (*p* < 0.001), reduced propionic acid (PCP: *p* < 0.001, INL: *p* < 0.01), and caused only slight changes in butyric acid. Notably, the levels of acetic, propionic, and butyric acids, as well as total SCFAs, were comparable between PCP and INL treatments, suggesting that these interventions exerted similar overall effects on SCFA production. In the OB group, both PCP and INL also increased the acetic acid (PCP: *p* < 0.05, INL: *p* < 0.001) and total SCFAs (INL: *p* < 0.001) levels relative to BLK, accompanied by reductions in both propionic and butyric acids. PCP treatment increased acetic acid and total SCFA levels in the OB group, demonstrating its effectiveness in modulating SCFA production, although was weaker compared to INL. In addition to SCFAs, several organic acids were also determined (Figure 2g–j). In the CON and OB groups, lactic acid increased after the PCP and INL treatments compared to BLK, whereas PCP treatment significantly reduced the levels of fumaric acid (CON: *p* < 0.001, OB: *p* < 0.01), malic acid (*p* < 0.01), and pyruvic acid (CON: *p* < 0.05).

With respect to tryptophan metabolism, the tryptophan level saw a decrease in both the CON and OB groups (Figure 2k). Meanwhile, indolelactic acid, a microbial-derived metabolite of tryptophan, was significantly elevated after PCP treatment in both the CON and OB groups (CON: *p* < 0.05, OB: *p* < 0.001) (Figure 2l).

### 3.5. Correlation Between Gut Microbiota Composition and Targeted Metabolites

**Figure 3:**
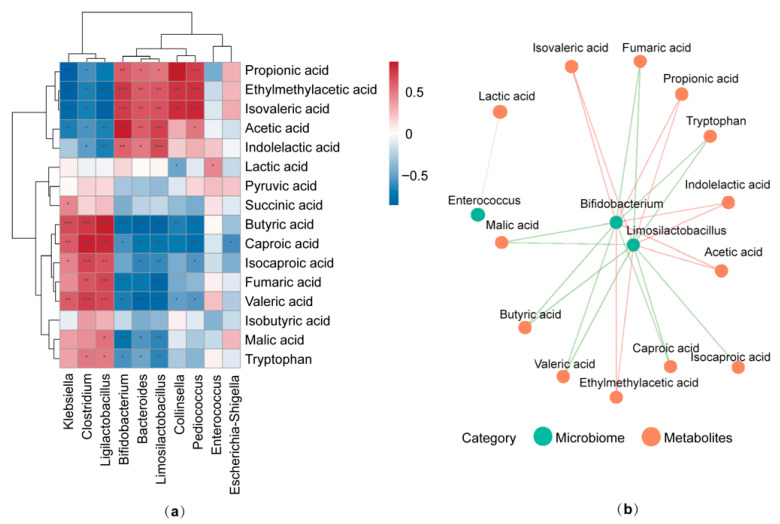
(**a**) Spearman correlation analysis between the gut microbial genera and metabolites. The heatmap shows correlations between the top 10 most abundant genera and representative metabolites. (**b**) The correlation network highlights key genera and metabolites, with orange and green edges denoting positive and negative associations, respectively. Statistical significance is indicated by * *p* < 0.05, ** *p* < 0.01, and *** *p* < 0.001.

Spearman correlation analysis between the relative abundance of the top ten gut microbiota and the concentration of metabolites was performed using the comprehensive dataset encompassing all groups (OB and CON) (Figure 3). *Bifidobacterium* and *Limosilactobacillus* were positively linked to acetic, ethylmethylacetic, propionic, and indolelactic acids, but negatively associated with butyric acid, certain other organic acids, and tryptophan. In addition, *Enterococcus* exhibited a positive correlation with lactic acid. Collectively, these findings revealed a strong link between gut microbiota and the generation of SCFAs and other metabolites, demonstrating that PCP can affect the gut microbiota composition and associated metabolic outputs.

### 3.6. Effects of PCP in Single-Strain Cultures

**Figure 4:**
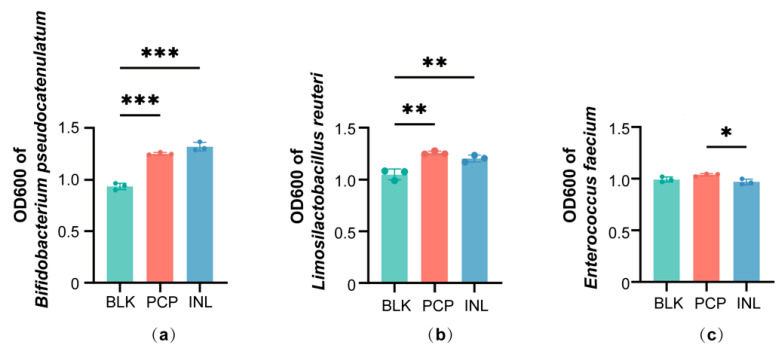
Effects of PCP on single-strain fermentation. OD600 of (**a**) *Bifidobacterium pseudocatenulatum*, (**b**) *Limosilactobacillus reuteri*, and (**c**) *Enterococcus faecium* after 24 h of co-culture. Data are expressed as the mean ± SD (n = 3), * *p* < 0.05, ** *p* < 0.01, *** *p* < 0.001.

16S rRNA sequencing identified shifts at the genus level after PCP treatment. To further validate these findings, we selected representative species for single-strain fermentation. The results showed that compared with BLK, both PCP and INL significantly promoted the growth of *Bifidobacterium pseudocatenulatum* (*p* < 0.001) and *Limosilactobacillus reuteri* (*p* < 0.01) after 24 h of co-culture (Figure 4a,b). For *Bifidobacterium pseudocatenulatum*, INL showed a slightly higher trend than PCP. In contrast, for *Limosilactobacillus reuteri*, PCP tended to promote greater growth than INL. In addition, *Enterococcus faecium* exhibited a slight increase after PCP treatment compared to BLK (Figure 4c).

## 4. Discussion

This study examined how PCP affected gut microbiota in normal-weight and obese children using in vitro fermentation. Our results indicated that PCP exerted significant regulatory effects on gut microbial composition and metabolic activities, highlighting its potential to contribute to microbiota balance and promote metabolic well-being in children.

In the CON group, the enrichment of *Bifidobacterium* and *Limosilactobacillus* was observed after PCP treatment, in agreement with previous studies in healthy adults showing that PCP enhanced these genera [2]. The genera are producers of SCFAs, and are involved in immune and barrier regulation, which may partially explain the observed metabolic shifts [25,26,27,28]. The reduction in *Escherichia-Shigella* is consistent with studies showing that increased SCFA levels and lower pH suppress Enterobacteriaceae such as *Escherichia-Shigella* [29]. Functionally, acetic acid not only represents a key end-product of *Bifidobacterium* and *Limosilactobacillus* metabolism but can also be cross-fed to butyrate-producing bacteria, thereby further contributing to gut health [30]. Interestingly, the marked reduction in intermediate metabolites such as fumaric acid, malic acid, and pyruvic acid has rarely been reported in PCP studies. Previous PCP studies have focused primarily on SCFA end-products [2]. The changes of intermediate metabolites suggested that PCP redirected microbial metabolism from incomplete fermentation pathways toward the highly efficient core anaerobic microbial fermentation pathways. This led to promoting the production of primary fermentation acids rather than the buildup of intermediate metabolites.

In the OB group, PCP also showed beneficial effects, though less pronounced than in the CON group. Da Silva et al. [31] reported that children with obesity had reduced gut microbiota diversity, and at the genus level, obese children also exhibited a significantly lower abundance of *Bifidobacterium*. According to the framework of Patlok et al. [32], genera with higher initial abundance expand more strongly when fiber is supplied, suggesting that the lower baseline level of PCP-responsive genera in obese children contributed to their attenuated response. PCP consistently promoted *Bifidobacterium*, indicating that its prebiotic effect on this genus showed stability across host conditions. Interestingly, *Enterococcus* decreased in the CON group but expanded in the OB group after PCP treatment, likely due to baseline community differences: in the CON group, expansion of *Bifidobacterium* and *Limosilactobacillus* limited *Enterococcus*, whereas in the OB group, *Enterococcus* more effectively utilized PCP-derived substrates [27]. *Enterococcus* is often considered an opportunistic pathogen and can be associated with dysbiosis and inflammation. However, *Enterococcus* also participates in lactic acid production and possesses anti-oxidant properties, indicating that its expansion may represent a functional adaptation in obese children [28,33,34]. Another intriguing finding is that ACE and Shannon diversity indices showed opposite trends after the PCP and INL treatments. This outcome suggests that while PCP successfully diversified the community by increasing the total number of unique species (richness, measured by ACE), it also strongly favored the expansion and dominance of a few specific beneficial taxa, thereby reducing the overall mathematical evenness (measured by Shannon) [35]. The differences between the CON and OB groups highlighted the complexity of the microbiota community in obese children. Overall, PCP maintained its prebiotic effect in the OB group by promoting *Bifidobacterium* and acetic acid, although with lower efficiency under this microbial background.

Interestingly, one metabolic feature was enhanced by PCP in both the CON and OB groups. PCP fermentation reduced tryptophan and raised indolelactic acid, an indole derivative produced from tryptophan mainly by *Bifidobacterium* and *Limosilactobacillus* [36,37,38]. Indolelactic acid functions as a natural ligand of the aryl hydrocarbon receptor (AhR). By activating AhR signaling, indolelactic acid has been shown to strengthen intestinal barrier integrity and promote mucosal immune regulation [39]. Because obesity is often characterized by the reduced production of beneficial tryptophan-derived metabolites, the PCP-induced increase in indolelactic acid suggests that PCP may help restore this impaired pathway and provide benefits beyond SCFA-mediated effects [40,41].

A seemingly counterintuitive finding was the reduction in propionate and the lack of butyrate production in the CON group, coupled with a negative correlation between *Bifidobacterium* and butyric acid across all samples. Similarly, butyrate production was reduced after PCP treatment in the OB group. Similar reductions in butyric acid have also been reported in other in vitro PCP fermentation studies [2]. This likely reflects the limitation of the in vitro batch fermentation model, which does not mimic the human colon’s complex bacterial cross-feeding. Rapid fermentation by bacteria like *Bifidobacterium* and *Limosilactobacillus* can lead to acetic and lactic acid accumulation in a 24 h closed-batch system. This buildup lowers the pH and inhibits the growth and metabolic activity of the more sensitive, butyrate-producing bacteria. In addition, −80 °C storage may further reduce the viability of butyrate-producing bacteria. Bircher et al. [42] reported that butyrate-producing species, such as *Roseburia intestinalis* and *Eubacterium hallii*, exhibit extremely low recovery rates after −80 °C storage. Although the reduced recovery of butyrate-producing bacteria during −80 °C storage may have lowered the absolute butyrate levels, this systematic effect applied equally across all treatments and does not affect the comparative conclusions. Therefore, the model likely captures the primary fermentation surge but fails to replicate the stable, continuous environment of the colon where these cross-feeding pathways would continuously convert acetate into butyrate [43].

In addition, at the community level, INL more effectively promoted *Bifidobacterium*, whereas PCP more strongly increased *Limosilactobacillus*. These findings are consistent with prior evidence showing that inulin exerts a characteristic bifidogenic effect and is preferentially utilized by *Bifidobacterium*, while PCP can be metabolized by *Limosilactobacillus* and has been shown to enhance their growth or activity [3,44]. Although direct comparisons between INL and PCP remain scarce, our observations align with these substrate preference trends. Single-strain fermentations further confirmed the community-level findings. With respect to SCFA production, INL exerted stronger effects than PCP. Beyond SCFAs, differences were also observed in tryptophan metabolism: INL induced a higher indolelactic acid level in the CON group, while PCP more strongly enhanced the indolelactic acid level in the OB group. Our findings show that INL and PCP have largely similar, yet possibly complementary effects, likely operating through overlapping mechanisms with minor differences in microbiota and metabolic pathways.

## 5. Conclusions

In summary, PCP exhibited clear prebiotic potential by modulating gut microbiota and regulating key metabolites in children. These findings support its potential application as a functional food intervention to improve gut health in both obese and normal-weight children. The benefits appeared more pronounced in normal-weight children, suggesting that differences associated with health status may influence the response. This is a preliminary in vitro finding of PCP’s effects. However, the findings must be interpreted with caution. The use of a small, (n= 5 per group), pooled in vitro model limits the statistical power and prevents analysis of inter-individual variation. A further limitation is the use of 16S rRNA gene sequencing, which detects DNA from both viable and non-viable cells. This method cannot confirm whether the detected microbes were metabolically active at the time of sampling. In vivo studies are required to confirm safety and efficacy, particularly in the obese pediatric population. Future studies should also investigate whether personalized interventions, including the potential combination of PCP with probiotics, could optimize its prebiotic efficacy, particularly in obese children.

## Data Availability

The original contributions presented in this study are included in the article/Supplementary Materials. Further inquiries can be directed to the corresponding authors.

## Supplementary Materials

The following supporting information can be downloaded at: https://www.mdpi.com/article/10.3390/foods14234077/s1, Table S1: Characteristics of subjects. Table S2: Content of monosaccharides in PCP. Figure S1: Monosaccharide content in PCP.

## Abbreviations

The following abbreviations are used in this manuscript:

ACE	Abundance-based Coverage Estimator
AhR	Aryl hydrocarbon receptor
ALT	Alanine aminotransferase
AST	Aspartate aminotransferase
BMI	Body mass index
FBG	Fasting blood glucose
FINS	Fasting insulin
HDL-C	High-density lipoprotein cholesterol
HOMA-β	Homeostasis model assessment of β-cell function
HOMA-IR	Homeostasis model assessment of insulin resistance
INL	Inulin
LDL-C	Low-density lipoprotein cholesterol
LEfSe	Linear Discriminant Analysis Effect Size
OTUs	Operational taxonomic units
PCoA	Principal coordinate analysis
PCP	Poria cocos polysaccharides
PLS-DA	Partial least squares discriminant analysis
SCFAs	Short-chain fatty acids
SCR	Serum creatinine
SD	Standard deviation
TC	Total cholesterol
TFA	Trifluoroacetic acid
TG	Triglycerides
UA	Uric acid
WGOC	Working Group on Obesity in China
