# Supplementary material for: In Vitro Assessment of the Prebiotic Effects of Poria Cocos Polysaccharides Using Fecal Microbiota from Normal-Weight and Obese Children
Source: Foods. 2025 Nov 27;14(23):4077. doi: 10.3390/foods14234077 (PMC12692152; doi:10.3390/foods14234077)
Supplement: Supplementary file 1 [file foods-14-04077-s001.zip › foods-3995430-supplementary.pdf]

Table S1. Characteristics of Subjects.

	CON GROUP(n=5)	OB GROUP(n=5)	P value
Male, n (%)	4(80.00%)	4(80.00%)	1.000
Age (years)	10.3±1.99	10.5±1.37	0.916
BMI (kg/㎡)	17.08±1.81	28.72±3.04	0.008
FBG (mmol/L)	4.91±0.24	4.86±0.41	0.917
FINS (μIU/ml)	7.67±4.07	37.22±30.77	0.028
HOMA-β	103.88±49.45	642.33±547.16	0.028
HOMA-IR	1.70±0.93	7.93±6.73	0.028
ALT (IU/L)	10.15±1.57	38.13±28.30	0.009
AST (IU/L)	23.40±5.39	30.04±16.17	0.917
SCR (μmol/L)	45.50±5.64	40.60±7.60	0.347
UA (μmol/L)	272.80±121.39	403.52±148.43	0.117
TC (mmol/L)	4.34±0.91	4.44±0.66	0.917
TG (mmol/L)	0.58±0.13	1.25±0.32	0.009
HDL-C (mmol/L)	1.38±0.29	1.38±0.26	0.917
LDL-C (mmol/L)	2.44±0.53	2.55±0.70	0.754

[1] **Table S1.** Data are expressed as mean±SD. Student's t test utilized for normally distributed contin uous variables, while the Wilcoxon rank-sum test was utilized for non-normally distributed continuo us variables. Chi-squared test was utilized for categorical variables. Abbreviation: BMI, body mass in dex; FBG, fasting blood glucose; FINS, fasting insulin; HOMA-β, homeostasis model assessment of β-cell function; HOMA-IR, homeostasis model assessment of insulin resistance; ALT, alanine aminotran sferase; AST, aspartate aminotransferase; SCR, serum creatinine; UA, uric acid; TC, total cholesterol; TG, triglycerides; HDL-C, high-density lipoprotein cholesterol; LDL-C, low-density lipoprotein cholest erol.

Table S2. Content of monosaccharides in PCP.

Monosaccharides	Fucose	Galactose	Glucose	Mannose
Retention Time(min)	4.417	12.650	14.584	18.392
Peak Area	0.451	1.731	28.127	1.064
Molar ratio	0.0197	0.0502	0.8974	0.0327

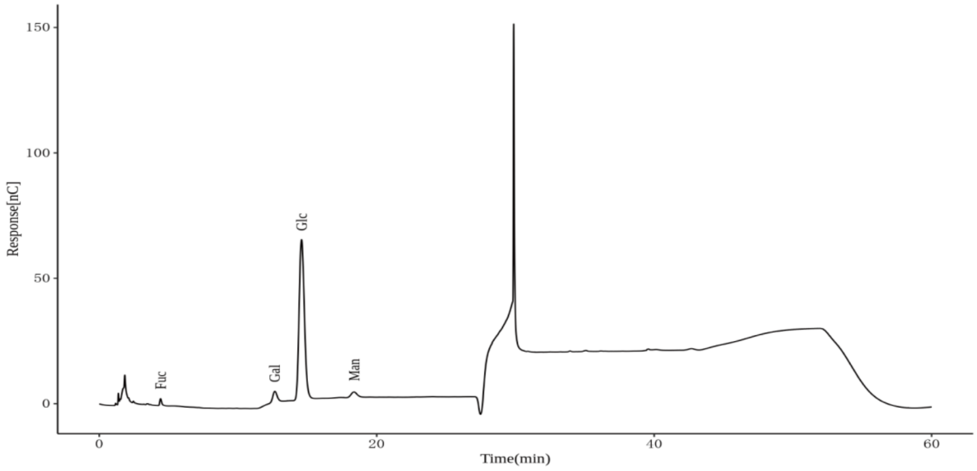

(**a**)

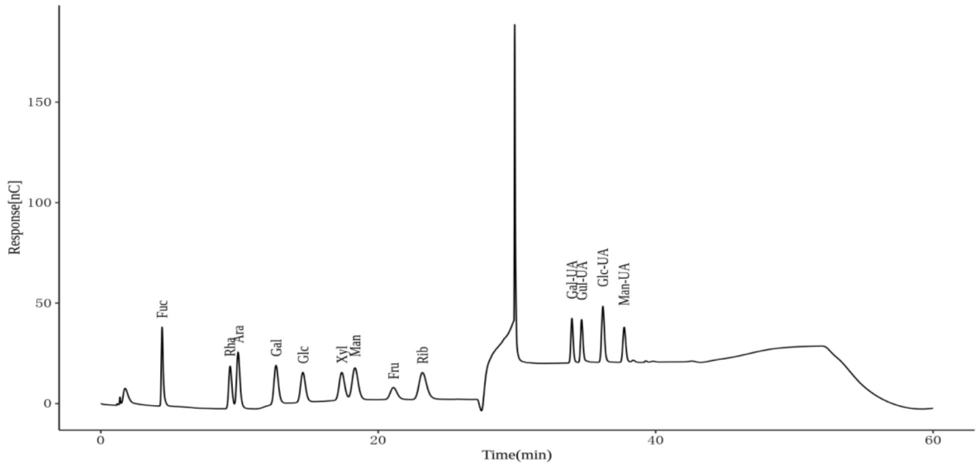

(**b**)

Figure S1. Monosaccharide content in PCP. (**a**) Ion chromatograms of PCP. (**b**) Ion chromatograms of the reference standard.
